# Expression of the p66Shc protein adaptor is regulated by the activator of transcription STAT4 in normal and chronic lymphocytic leukemia B cells

**DOI:** 10.18632/oncotarget.10977

**Published:** 2016-08-01

**Authors:** Francesca Cattaneo, Laura Patrussi, Nagaja Capitani, Federica Frezzato, Mario Milco D'Elios, Livio Trentin, Gianpietro Semenzato, Cosima T. Baldari

**Affiliations:** ^1^ Department of Life Sciences, University of Siena, Siena, Italy; ^2^ Department of Experimental and Clinical Medicine, University of Florence, Firenze, Italy; ^3^ Department of Medicine, University of Padua, Padova, Italy

**Keywords:** transcriptional regulation, p66Shc, STAT4, lisofylline, CLL

## Abstract

p66Shc attenuates mitogenic, prosurvival and chemotactic signaling and promotes apoptosis in lymphocytes. Consistently, p66Shc deficiency contributes to the survival and trafficking abnormalities of chronic lymphocytic leukemia (CLL) B cells. The mechanism of *p66shc* silencing in CLL B cells is methylation-independent, at variance with other cancer cell types. Here we identify STAT4 as a novel transcriptional regulator of p66Shc in B cells. Chromatin immunoprecipitation and reporter gene assays showed that STAT4 binds to and activates the *p66shc* promoter. Silencing or overexpression of STAT4 resulted in a co-modulation of p66Shc. IL-12-dependent STAT4 activation caused a coordinate increase in STAT4 and p66Shc expression, which correlated with enhanced B cell apoptosis. Treatment with the STAT4 inhibitor lisofylline reverted partly this effect, suggesting that STAT4 phosphorylation is not essential for but enhances *p66shc* transcription. Additionally, we demonstrate that CLL B lymphocytes have a STAT4 expression defect which partly accounts for their p66Shc deficiency, as supported by reconstitution experiments. Finally, we show that p66Shc participates in a positive feedback loop to promote STAT4 expression. These results provide new insights into the mechanism of p66Shc expression in B cells and its defect in CLL, identifying the STAT4/IL-12 pathway as a potential therapeutic target in this neoplasia.

## INTRODUCTION

p66Shc is a member of the Shc (Src homology 2 domain containing) family of protein adaptors that negatively regulates proliferative and pro-survival signaling [1–6] and promotes moreover apoptosis by increasing intracellular oxidants [7]. As opposed to the ubiquitously expressed p52Shc and p46Shc, which are encoded by the same locus, p66Shc is expressed in a tissue-specific manner due to the presence of two alternative promoters in the *ShcA* locus regulating the transcripts encoding p52Shc/p46Shc and p66Shc, respectively [8]. The regulatory region of *p66shc* is characterized by the presence of a CpG-rich region that can be hyper-methylated, leading to promoter silencing [8, 9]. Although DNA modifications are responsible for *p66shc* silencing in epithelial as well as in T cells, the mechanism of p66Shc regulation in other cell types has yet not been elucidated. The absence of transcription factors specifically able to bind and activate the *p66shc* promoter may provide an alternative or additional mechanism, as exemplified by nuclear erythroid 2-related factor 2 (Nrf2), which binds to an antioxidant response element on the *p66shc* promoter [10, 11].

We have recently shown that neoplastic B cells from Chronic Lymphocytic Leukemia (CLL) patients exhibit a defect in *p66shc* expression, with the lowest levels displayed by patients with unfavorable prognosis [6]. Interestingly, although the presence of methylated CpG sites in the *p66shc* promoter may account in part for the relatively low expression levels of p66Shc in healthy B cells, neither the overall methylation status of the CpG-rich region nor the methylation of individual CpG sites differ between healthy and CLL B cells [6], indicating that a transcriptional rather than epigenetic mechanism may account for the p66Shc expression defect in neoplastic cells.

Here we show that STAT4 regulates p66Shc expression in B cells by interacting with several specific binding sites in the *p66shc* promoter. Of note, the p66Shc defect in CLL B cells correlates with impaired STAT4 expression. Interestingly, we found that p66Shc is in turn able to promote the expression of several genes participating in the IL-12 pathway and regulated by STAT4, including STAT4 itself, and reconstitution of p66Shc in CLL B cells normalizes the levels of STAT4. The data highlight a new mechanism of transcriptional regulation of p66Shc in B cells mediated by STAT4 binding to the *p66shc* promoter and provide evidence of a feedback regulatory loop whereby p66Shc modulates STAT4. They identify moreover STAT4 deficiency as a potential player in the response of CLL B cells with the tumor microenvironment.

## RESULTS AND DISCUSSION

### Gene expression profile analysis associates p66Shc to expression of IL-12 responsive genes in B cells

We have shown that p66Shc is able to modulate the expression of several genes critical to B-cell survival and homing through both its adaptor and pro-oxidant activities [6, 12]. To achieve insights into the processes regulated by p66Shc we used an unbiased approach involving a gene expression profile analysis on B cells stably transfected with a plasmid encoding p66Shc (MEC-p66) or the respective empty vector (MEC-Ctr). The MEC-1 cell line was used for these experiments as endogenous *p66shc* is completely silenced by promoter methylation, as supported by the fact that treatment with the demethylating agent 5-Aza-2′-deoxycytidine (AZA, decitabine) restored its expression (Supplementary Figure S1A) [13]. Two independent mRNA extractions were profiled for each sample using the Affymetrix HuGene 2.0-st-v1 array. An ANOVA model to identify genes differentially expressed between the two groups was created and the transcripts with a fold-change higher than 2 and a statistically significant *p*-value (*P*<0.05) were identified. A hierarchical clustering of the results is shown in Supplementary Figure S2.

**Figure 1:**
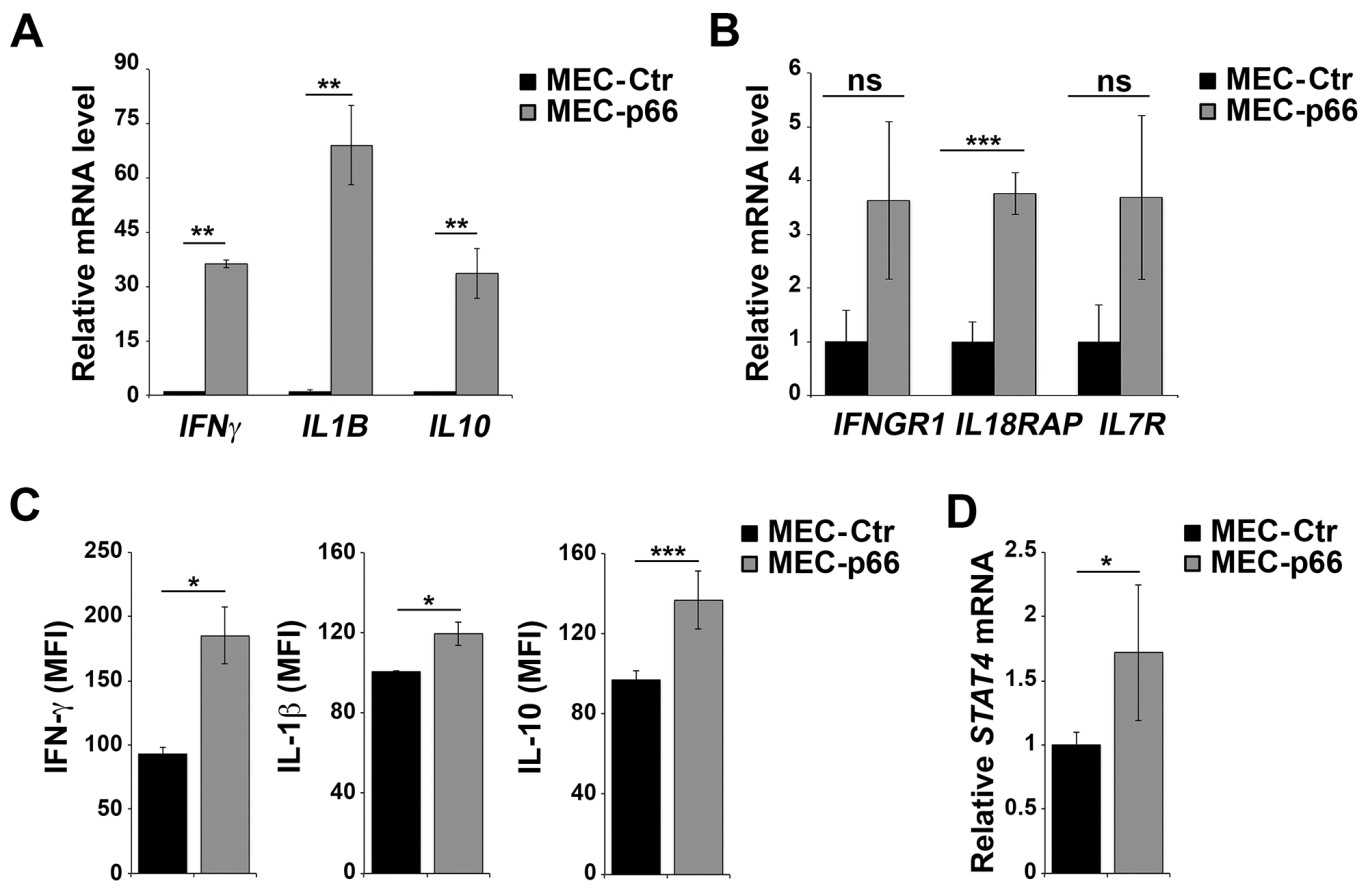
p66Shc alters the expression of several genes linked to the IL-12 pathway in B cells **A, B, D.** qRT-PCR analysis of *IFNγ, IL1B, IL10, IFNGR1, IL18RAP, IL7R* and *STAT4* mRNA in MEC-1 cells stably transfected with a construct encoding p66Shc (MEC-p66) or empty vector (MEC-Ctr). The relative abundance of gene transcripts was determined on triplicate samples from ≥3 independent mRNA extractions using the ΔΔCt method and is expressed as normalized fold expression (mean±SD). **C.** Flow cytometric analysis of intracellular IFN-γ, IL-10 and IL-1β in MEC-Ctr and MEC-p66 cells treated for 24 h with a combination of PMA and A23187 in the presence of brefeldin A. Data are expressed as mean fluorescence intensity (MFI) ± SD (n≥3). ***P≤0.001, **P≤0.01, *P≤0.05.

Six genes, *IL10*, *IL18RAP*, *IL1B, IL7R, IFNγ*, and *IFNGR1*, were chosen for further validation based on their mRNA fold-changes and *p*-values (Table 1). Validation was carried out by qRT-PCR on three independent mRNA extractions from MEC-p66 and MEC-Ctr cells. The levels of *IFNγ*, *IL1β* and *IL10* (Figure 1A), as well as of *IFNγR1*, *IL18RAP* and *IL7R* (Figure 1B), mRNA, were confirmed to be up-regulated in p66Shc-overexpressing cells compared to the empty vector transfectant. Consistent with the qRT-PCR data, IFN-γ, IL-1β and IL-10, whose mRNA levels showed the largest fold-changes, were up-regulated in MEC-p66 cells compared to control cells, as assessed by flow cytometry (Figure 1C).

**Table 1:** List of IL-12 regulated genes found to be differentially up-regulated in p66Shc-overexpressing MEC-1 cells versus control

Gene Symbol	Gene name	Fold-Change	*p*-Value
***IL10***	Interleukin-10	12.7101	0.000006
***IL18RAP***	Interleukin-18 Receptor Accessory Protein	4.3073	0.001364
***IL1B***	Interleukin-1β	3.3917	0.001643
***IL7R***	Interleukin 7 receptor α chain	2.8143	0.000170
***IFNγ***	Interferon γ	2.5490	0.000992
***IFNGR1***	Interferon-γ Receptor α chain	2.0731	0.000330

Data obtained from the analysis of the Affymetrix HuGene-2.0-st-v1 arrays using Partek Genomics Suite version 6.6 software.

Interestingly, the products of the six genes identified in the analysis participate in pathways driven by IL-12 and its primary mediator, Signal Transducer and Activator of Transcription 4 (STAT4). The IL-12 pathway is essential for CD4^+^ T cell commitment to the Th1 subset [14, 15], where IL-12 promotes the STAT4-dependent expression of IFN-γ [16, 17]. The genes of the IL-12/IFN-γ axis include *IFNGR1*, *IL18RAP*, *IL10*, *IL1B* and *IL7R* [18–21]. Since STAT4 expression and activation are crucial for the response to IL-12 [22], the levels of STAT4 were quantified in p66Shc overexpressing MEC-1 cells. As shown in Figure 1D, the STAT4 transcript was significantly up-regulated in MEC-p66 cells. This gene had not been identified in the Affymetrix array analysis because the difference compared to the MEC-Ctr (1.72-fold) was lower than the 2-fold threshold used.

### STAT4 recognizes and binds to the *p66shc* regulatory region in B cells

At variance with other cell types [8, 9, 23–25], gene promoter modification have been ruled out as the primary mechanism of transcriptional regulation of *p66shc* in both normal and CLL B cells notwithstanding a partial methylation of the CpG sites [6], indicating that different mechanisms are operational in these cells, such as recruitment of chromatin-remodeling enzymes or availability of specific transcription factors.

To identify transcriptional regulators we performed an *in silico* analysis using the JASPAR software on the *p66shc* locus, focusing on a region of ~2 kb encompassing nucleotides −1830 to +71, +1 being the transcription start site (TSS). We identified 1668 putative binding sites for several transcription factors in the sequence analyzed, confirming previously reported binding sequences for factors such as SP1, NF-AT and GATA3 [8]. Interestingly, we identified several sites for members of the STAT family, suggesting that these proteins might participate in *p66shc* regulation. We focused on STAT4, as this is the main effector in the IL-12 pathway that we found altered in p66Shc-overexpressing cells.

**Figure 2:**
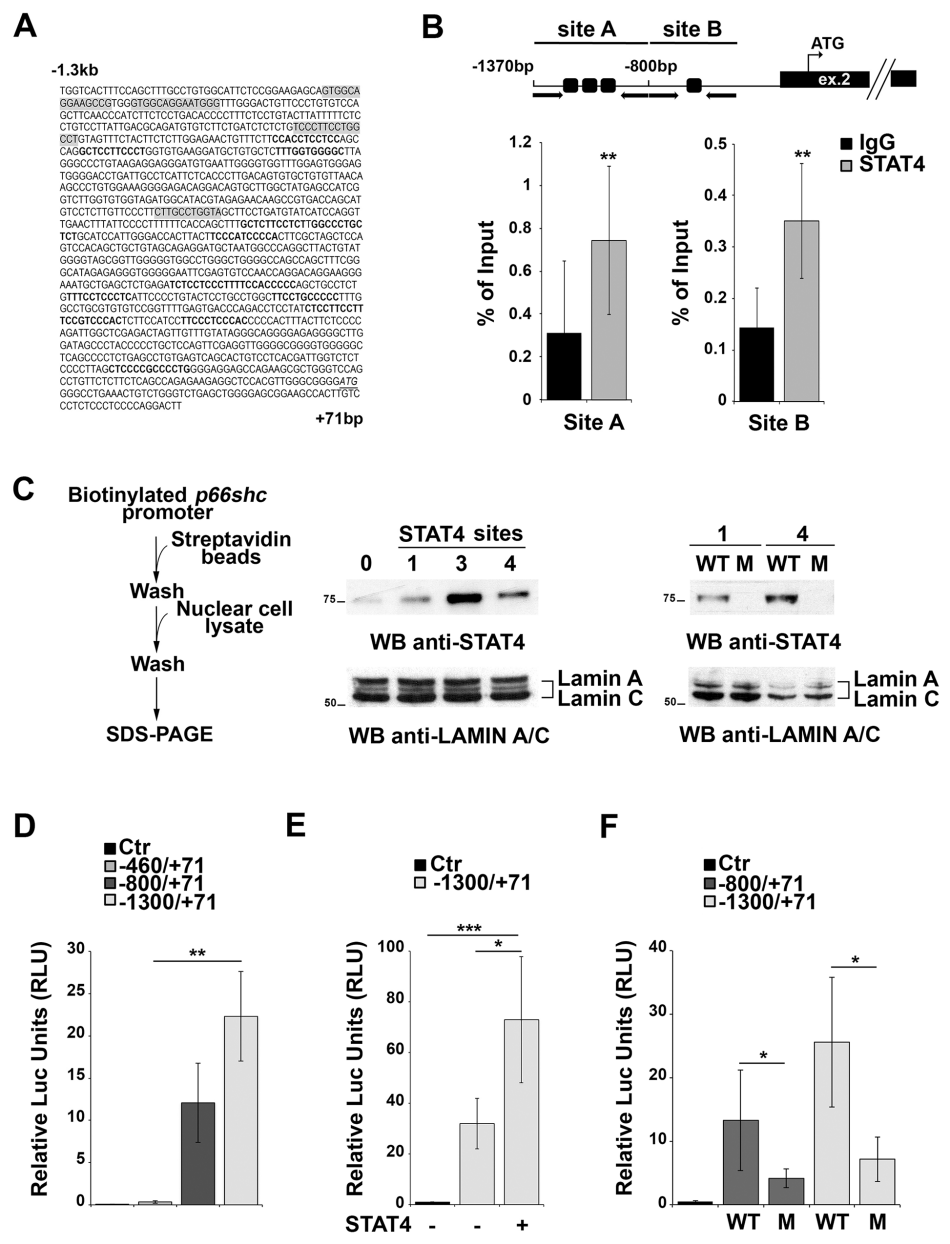
STAT4 regulates *p66shc* expression **A.** *In silico* promoter analysis of the human *p66shc* promoter highlighting several putative STAT4 binding sites (grey shaded regions). SP1 binding sites identified in the analysis and known to be present in the *p66shc* promoter are shown in bold. The transcription start site (ATG) is also shown (underlined, italic). **B.** *Top*, schematic representation of the *p66shc* region taken into consideration for the ChIP experiments. Black arrows indicate the position of the primers used, dark squares the position of the STAT4 binding sites. The *p66shc* TSS is located in exon 2. *Bottom*, Nuclear extracts of EBV-B cells were subjected to ChIP assay with an antibody specific for STAT4. Precipitated DNA was amplified by qRT-PCR using primers described above. Unspecific IgG was used as control. Data are presented as percentage of input DNA (mean±SD; n=6). **C.** Scheme of the *in vitro* binding experiments. EBV-B nuclear extracts were used to pull down STAT4 bound to the biotinylated *p66shc* promoter, and bound STAT4 was detected by immunoblot. Lanes 1, 3 and 4 correspond to the beads bound to the promoter region containing one, three or all four STAT4 binding sites, respectively. Streptavidin beads incubated with nuclear extract were used as negative control (lane 0). Pull-down experiment using mutants of the putative STAT4 binding sites (M) showed no STAT4 immunoreactive band compared to the control (WT). (n=3). **D-F.** *p66shc* promoter activation assays in EBV-B cells. **(D)** Cells were co-transfected with reporter plasmids carrying *p66shc* promoter sequences containing none (pGL4p66Shc-460/+71), one (pGL4p66Shc-800/+71), or all (pGL4p66Shc-1300/+71) STAT4 binding sites, or empty vector (Ctr). A co-transfected *Renilla* plasmid was used as normalization control. **(E)** EBV-B cells were co-transfected with the pGL4p66Shc-1300/+71 luciferase reporter, the *Renilla* transfection control and a plasmid encoding STAT4. **(F)** EBV-B cells were co-transfected with plasmids containing the mutated STAT4-binding sequences or the corresponding WT sequences, and the *Renilla* transfection control. ***P≤0.001; **P≤0.01, and *P≤0.05 (n=3)

Four putative binding sites for STAT4 were identified in the proximity of the *p66shc* TSS (Figure 2A, Supplementary Table S1). These sites are far from the CpG-rich region, suggesting that they may be accessible even when the CpG sites are partially methylated. Chromatin Immunoprecipitation (ChIP) assays were performed to assess whether STAT4 can bind to these sites. For these experiments we used EBV-immortalized primary human B cells rather than MEC-1 cells because, as opposed to the latter, these cells express p66Shc (Supplementary Figure S1B), indicating that the *p66shc* promoter is accessible to transcription factors. Two sets of primers were designed to amplify the regions of interest, one spanning nucleotides −1370 to −807 containing three clustered STAT4 binding sites, the other nucleotides −800 to −512 covering the single STAT4 site most proximal to the *p66shc* TSS (site A and B, respectively; Figure 2B). STAT4 binding was evaluated by ChIP assays using anti-STAT4 IgG or control non-specific IgG followed by qRT-PCR of the regions of interest, and normalized to the input (Figure 2B). A statistically significant binding of both site A and site B was observed when the ChIP assay was carried out using the anti-STAT4 antibody compared to the IgG control (Figure 2B).

STAT4 binding to the *p66shc* promoter was additionally confirmed using *in vitro* pull-down assays. The regions of interest, containing one, three or all the STAT4 binding sites, were amplified by PCR, using a 3′ modified primer to obtain a biotinylated *p66shc* promoter. The PCR products were bound to streptavidin beads and then incubated with nuclear cell extracts from EBV-B cells, then washed and subjected to SDS-PAGE. The presence of STAT4 bound to the promoter fragments was detected using an anti-STAT4 antibody. An immunoreactive band, corresponding to STAT4, was present in the lanes containing the beads incubated with the PCR fragments, only a low background signal being detectable in the control sample incubated with streptavidin beads only (Figure 2C). No STAT4 was pulled down when biotinylated fragments containing one or all four STAT4 binding sites mutated were used, confirming that the putative STAT4 binding sites on the *p66shc* promoter are actually able to bind STAT4 (Figure 2C). Collectively, these results identify STAT4 as a novel transcription factor able to associate with the *p66shc* promoter.

### STAT4 activates the *p66shc* promoter

To assess whether the presence of STAT4 binding sites influences the activity of the *p66shc* promoter *in vitro* EBV-B cells were transfected with luciferase reporter constructs containing fragments of the *p66shc* promoter differing in the number of STAT4 binding sites, i.e. none (pGL4p66Shc-460/+71), one (pGL4p66Shc-800/+71), or all four binding sites (pGL4p66Shc-1300/+71). The presence of one STAT4 site was sufficient to enhance *p66shc* promoter activity compared to the fragment with no STAT4 binding site, with a further increase in the presence of the pGL4p66Shc-1300/+71 construct, containing four STAT4 binding sites (Figure 2D). These data indicate that at least one accessible STAT4 binding site in the *p66shc* promoter is required to promote transcription.

EBV-B cells were next co-transfected with the pGL4p66Shc-1300/+71 reporter and a STAT4-encoding plasmid to verify whether increasing STAT4 levels would result in an enhancement in the *p66shc* promoter activity compared to the respective empty vector control. An increase in luciferase activity was observed in co-transfected cells, indicating that STAT4 promotes *p66shc* transcription (Figure 2E). Of note, EBV-B cells tranfected with *p66shc* promoter constructs harboring point mutations in one or all the STAT4 binding sites displayed a significant reduction in the levels of luciferase activity compared to cells transfected with the WT reporters (Figure 2F). Moreover the activity of the mutated *p66shc* promoter was not enhanced in cells co-transfected with the STAT4 encoding construct (0.96±0.03 fold compared to cells trasfected with empty vector), at variance with the increase observed with the WT reporter (>2 fold, see also Figure 2E). Hence STAT4 binding to its recognition sites on the *p66shc* promoter is required for p66Shc expression (Figure 2F).

**Figure 3:**
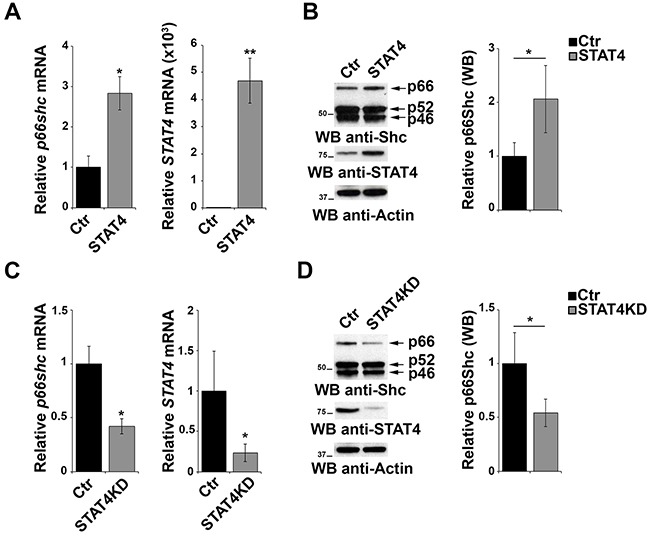
STAT4 modulates the levels of p66Shc in B cells **A.** qRT-PCR analysis of *STAT4* and *p66shc* mRNA in EBV-B cells transiently transfected with either empty vector (Ctr) or an expression construct encoding STAT4 (STAT4). **B.** Immunoblot analysis of p46Shc, p52Shc and p66Shc 48 h after transfection with the STAT4-encoding construct in EBV-B cells. The histogram shows the quantification of the p66Shc immunoreactive band (n≥3). **C.** EBV-B cells were transfected with esiRNA targeting *STAT4* and the levels of *p66shc* and *STAT4* were measured by qRT-PCR. The relative abundance of the gene transcripts was determined on triplicate samples from at least 3 independent experiments using the ΔΔCt method and is expressed as the normalized fold expression (mean±SD). **D.** Immunoblot analysis of p46Shc, p52Shc and p66Shc 48 h after transfection of EBV-B cells with esiRNA targeting *STAT4* (n≥3). Filters were reprobed for actin as loading control. The histogram shows the quantification of the p66Shc immunoreactive band. **P≤0.01; and *P≤0.05.

To evaluate the effect of STAT4 overexpression on endogenous p66Shc, EBV-B cells were transiently transfected with the STAT4-encoding plasmid and the levels of p66Shc were measured by qRT-PCR and immunoblot. p66Shc expression was increased both at the mRNA and protein level in cells overexpressing STAT4 compared to empty vector controls (Figure 3A, 3B). As a complementary experiment, STAT4 was knocked down by transfecting EBV-B cells with specific esiRNA. A reduction in *p66shc* mRNA and protein was observed under these conditions, supporting the notion that STAT4 is required to promote *p66shc* transcription (Figure 3C, 3D).

### IL-12 dependent STAT4 phosphorylation enhances *p66shc* promoter activity

**Figure 4:**
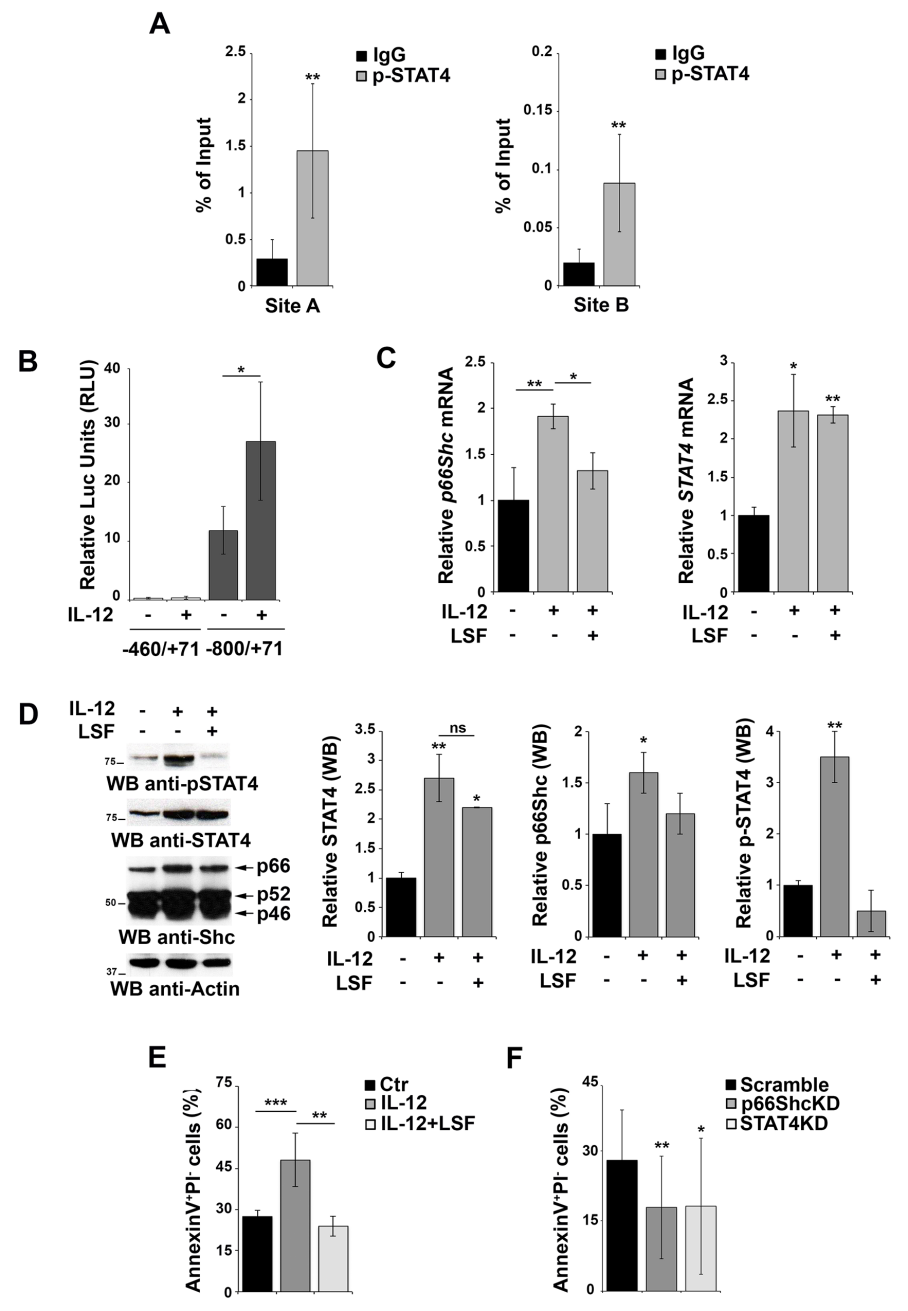
Activation of STAT4 promotes p66Shc transcription and B-cell apoptosis **A.** Nuclear extracts of EBV-B cells were subjected to ChIP assay with an antibody specific for p-STAT4. Precipitated DNA was amplified by qRT-PCR. Unspecific IgG was used as control. Data are presented as percentage of input DNA (mean±SD; n=6). **B.** Reporter gene assays on EBV-B cells co-transfected with the *p66shc* promoter reporter plasmid (pGL4p66Shc-460/+71) containing one STAT4 site and the control *Renilla* plasmid. Cells were stimulated or not with the IL-12 for 3 h prior to measuring luciferase activity (n>3). **C.** qRT-PCR analysis of *p66shc* and *STAT4* mRNA in EBV-B cells as above in presence or absence of 20 μM LSF (n≥3). **D.** Immunoblot analysis of STAT4, pSTAT4 and the three Shc isoforms in EBV-B cells stimulated or not with IL-12 in presence or absence of 20 μM LSF. Filters were reprobed for actin as loading control. The histograms show the quantification of STAT4, p-STAT4 and p66Shc, normalized to actin (n=3). **E.** Flow cytometric analysis of AnnexinV^+^/PI^−^ EBV-B cells stimulated or not with IL-12 in presence or absence of 20 μM LSF followed by treatment with 500 ng/ml A23187. **F.** Flow cytometric analysis of AnnexinV^+^/PI^−^ EBV-B cells transfected with siRNAs targeting *p66shc* or *STAT4*, respectively, and stimulated with IL-12/A23187. Cells transfected with a scrambled siRNA were used as control. Data represent the mean±SD of the Annexin V^+^/PI^−^ cells (n=3). ***P≤ 0.001; **P≤0.01; and *P≤0.05.

Although STAT4 activity is regulated by IL-12-dependent phosphorylation, our reporter gene experiments suggest that the overexpression of the transcription factor itself might be sufficient to activate the *p66shc* promoter independently of its phosphorylation, consistent with the finding that also unphosphorylated STATs are able to shuttle between cytoplasm and nucleus to activate transcription [26]. A role for STAT4 phosphorylation can however also be hypothesized, as EBV-B cells have been shown to produce IL-12 [27]. To evaluate whether phosphorylated STAT4 binds more efficiently the *p66shc* promoter, we performed ChIP experiments in EBV-B cells using anti-p-STAT4 IgG and control non-specific IgG. The results obtained using the anti-pSTAT4 IgG (Figure 4A) were comparable to those obtained using the anti-STAT4 IgG (Figure 2B), suggesting that either STAT4 binds to its target sites with the same efficiency as the phosphorylated form or the STAT4 antibody is actually pulling down the phosphorylated form bound to the *p66shc* promoter.

To further address this issue and clarify the importance of IL-12 dependent STAT4 phosphorylation on the activity of the *p66shc* promoter, EBV-B cells were transfected with the p66Shc reporter construct carrying one STAT4 site (pGL4p66Shc-800/+71) and stimulated or not with IL-12 to promote STAT4 phosphorylation [28]. While significant levels of luciferase activity were observed in the absence of IL-12, these were enhanced in IL-12-treated cells (Figure 4B), indicating that IL-12-dependent STAT4 phosphorylation potentiates *p66shc* transcription in B cells. It is however noteworthy that IL-12 treatment not only induced STAT4 phosphorylation but also increased the basal levels of *STAT4* mRNA and protein (Figure 4C, 4D).

To elucidate the contribution of STAT4 phosphorylation to the IL-12-dependent increase in p66Shc expression we analyzed the response to IL-12 of EBV-B cells pretreated with lisofylline (LSF), an inhibitor of STAT4 phosphorylation. LSF treatment resulted in a reduction in the levels of phosphorylated STAT4 without affecting the overall mRNA and protein levels (Figure 4C, 4D), which allows to specifically dissect the role of STAT4 phosphorylation. As shown in Figure 4C and 4D, the IL-12 dependent increase in p66Shc expression was largely abrogated in the presence of LSF. These results indicate that IL-12 treatment enhances *p66shc* transcription by increasing the levels of the activated form of its transcription factor STAT4.

Cell treatment with LSF alone resulted in a decrease in the basal levels of phosphorylated STAT4, however no changes in the mRNA and proteins levels of p66Shc were observed at the lower drug concentrations used (Supplementary Figure S3). In the presence of higher LSF concentrations, which affected more severely the basal levels of STAT4 phosphorylation, a decrease in p66Shc was detected, albeit not statistically significant. Nevertheless this was paralleled by a decrease in the p52/p46 Shc isoforms (Supplementary Figure S3B), suggesting a general and unspecific effect of LSF treatment at that concentration. Collectively, these results indicate that unphosphorylated STAT4 is sufficient to promote *p66shc* transcription but that this process is potentiated when STAT4 is phosphorylated in response to IL-12.

Once bound to its recognitions sites STAT4 is able to modulate gene transcription by recruiting not only other transcription factors but also chromatin-remodeling enzymes to promote active epigenetic marks such as trimethylation of lysine 4 of histone 3, leading to increased gene transcription, as shown for Th cells [29]. The fact that *p66shc* expression could be restored in cells that normally do not express it by treatment not only with DNA demethylating agents but also with inhibitors of histone deacetylases [8] suggests a role for the “histone code”, which may involve STAT4, in the regulation of *p66shc* transcription.

p66Shc promotes apoptosis in response to oxidative stress [4, 5, 7]. To investigate the role of STAT4 on the pro-apoptotic activity of p66Shc, EBV-B cells were stimulated with IL-12, alone or in combination with LSF, and then treated with the Ca^2+^ ionophore A23187, a potent inducer of apoptosis. Flow cytometric analysis revealed an increase in the number of Annexin V^+^/propidium iodide^−^ cells following IL-12 stimulation, which was reversed by pre-treatment of the cells with LSF (Figure 4E). These results suggest, albeit indirectly, that activation of the IL-12 pathway leads to an increase in apoptosis as a result of the enhanced p66Shc expression observed under these conditions. To further test this hypothesis we measured the proportion of Annexin V^+^/propidium iodide^−^ cells after transfection with siRNAs targeting *p66shc* or *STAT4* and stimulated with IL-12 and A23187. Interestingly, a reduction in the level of apoptotic cells was observed in EBV-B cells lacking STAT4, suggesting that STAT4 is required for IL-12/A23187 induced apoptosis. A similar reduction was observed in cells in which p66Shc was silenced, further supporting the importance of p66Shc in stress-induced apoptosis in B cells (Figure 4F).

### p66Shc deficiency in CLL is associated with impaired STAT4 expression

**Figure 5:**
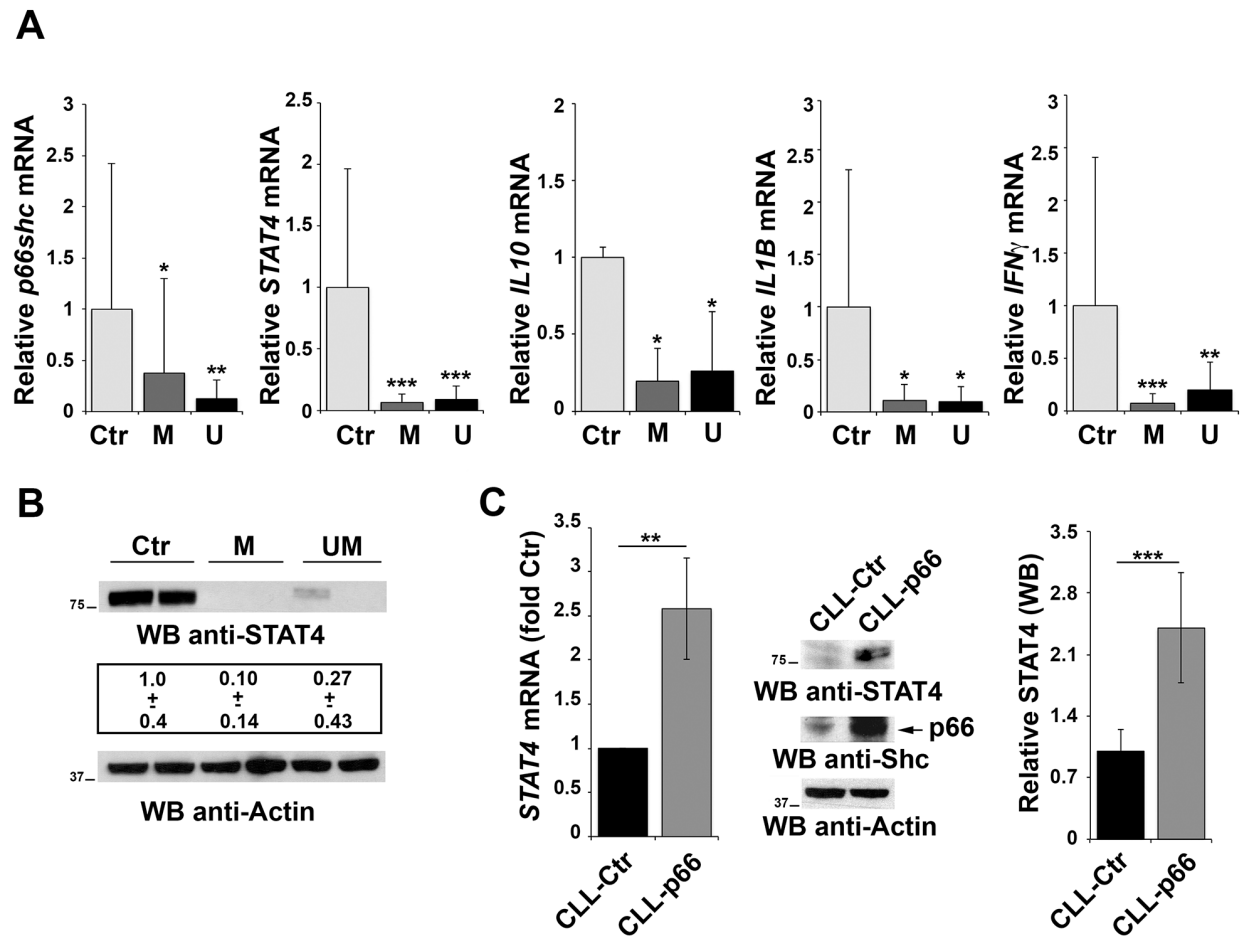
Impaired STAT4 expression in primary CLL B cells **A.** qRT-PCR analysis of *p66shc*, *STAT4*, *IL10*, *IL1β* and *IFNγ* mRNA in purified peripheral B cells from either healthy donors (Ctr, n=9) or CLL patients with mutated (M; n=15) or unmutated (U; n=15) *IGHV*. **B.** Representative immunoblot analysis of STAT4 in 2 healthy donors, 2 M-CLL and 2 U-CLL patients. The numbers below the representative blot refer to the quantification of STAT4 immunoreactive band in B-cell lysates from 10 M-CLL, 9 U-CLL and 6 healthy controls, of which at least 1 was included in each gel as reference. **C.** qRT-PCR analysis of *STAT4* mRNA in CLL B cells nucleofected with either empty vector (CLL-Ctr) or an expression construct encoding p66Shc (CLL-p66). The relative abundance of *STAT4* transcript was determined on triplicate samples from each patient using the ΔΔCt method and is expressed as the normalized fold expression (mean±SD; empty vector controls taken as 1 for all CLL samples). All samples (n=6) were checked for reconstitution of *p66shc* expression by qRT-PCR (data not shown). A representative immunoblot of STAT4 and p66Shc is shown on the right. Filters were reprobed for actin as loading control. The histogram shows the quantification of STAT4 in B-cell lysates from 3 reconstituted CLL samples. ***P≤ 0.001; **P≤0.01; and *P≤0.05.

We have previously reported that CLL B cells have a defect in *p66shc* expression that is not caused by methylation of the CpG dinucleotides in the gene promoter [6]. To understand whether STAT4 is involved in this defect, the relative abundance of the STAT4 transcript was measured in peripheral blood B cells from 30 CLL patients and 9 healthy controls. Patients were classified according to their mutational status of the Ig heavy chain variable (*IGHV*) region as mutated (M) or unmutated (UM), the latter being an unfavorable prognostic factor of disease progression and outcome [30]. CLL B cells showed a significant reduction in the levels of *STAT4* mRNA, as assessed by qRT-PCR, compared with healthy controls, independently of the *IGHV* mutational status (Figure 5A). STAT4 deficiency in CLL B cells was confirmed by immunoblot (Figure 5B), suggesting that the defect in *p66shc* expression in these patients might be accounted for, at least in part, by the low levels of STAT4. Consistent with the STAT4 defect, the mRNA levels of *IFNγ*, *IL10* and *IL1β* which are regulated by STAT4 [16, 19] were reduced in CLL compared to healthy B cells (Figure 5A).

### A positive feedback loop couples p66Shc to STAT4 expression

**Figure 6:**
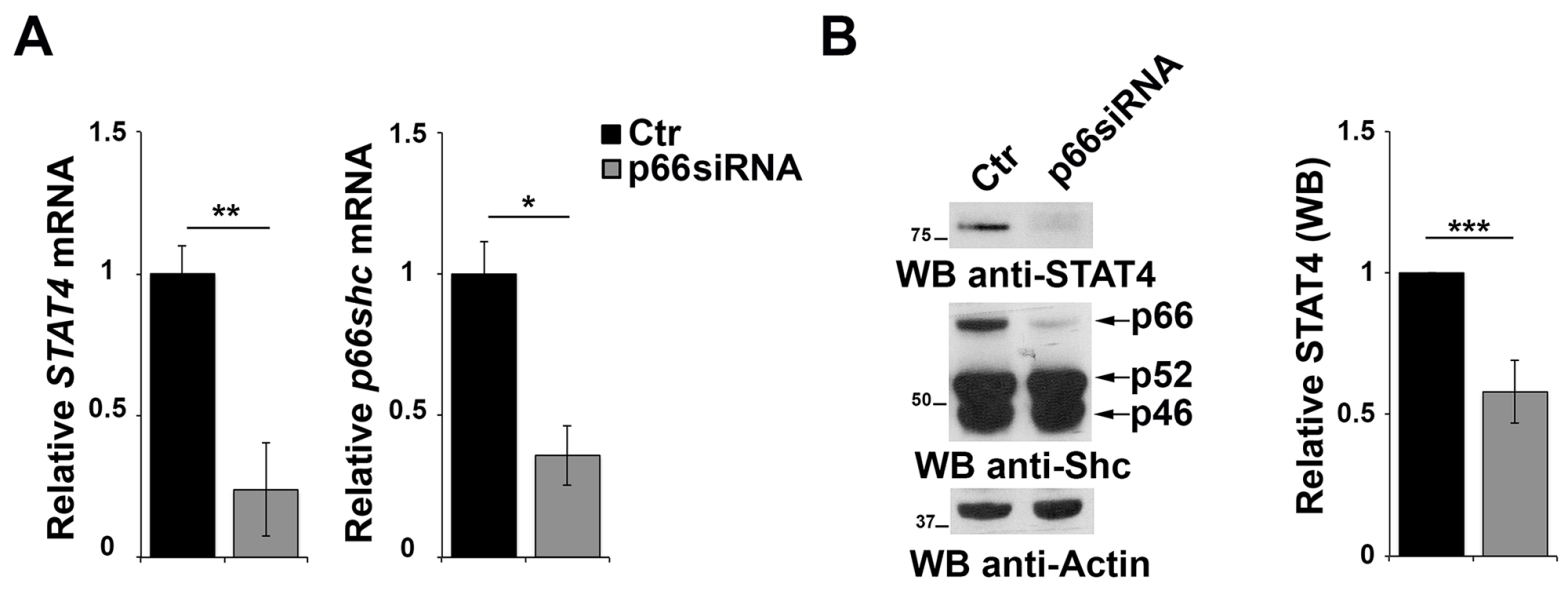
p66Shc affects STAT4 protein stability **A.** qRT-PCR analysis of *STAT4* and *p66shc* mRNA in EBV-B cells transfected with siRNA targeting *p66shc*. The relative abundance of the genes transcripts as described above and is expressed as the normalized fold expression (mean±SD, (n≥3). **B.** Immunoblot analysis of STAT4 and the three Shc isoforms in EBV-B cells transfected with a siRNA targeting *p66shc*. Filters were reprobed for actin as loading control. The histogram shows the quantification of the levels of STAT4, normalized to actin (n≥3). ***P≤0.001; and **P<0.01.

The results of the gene array data showing that p66Shc overexpression leads to an increase in the levels of STAT4 in MEC-1 cells suggest the existence of a regulatory feedback loop between STAT4 and p66Shc which could account for the STAT4 defect observed in CLL B cells. To test this hypothesis positing that p66Shc, which relies on STAT4 for its expression, is in turn implicated in STAT4 expression, CLL B cells were transfected with a p66Shc-encoding construct and the levels of STAT4 were measured by qRT-PCR and immunoblot. As shown in Figure 5C, reconstitution of p66Shc expression in CLL cells resulted in a significant increase in the levels of STAT4 (Figure 5C). This result suggests that p66Shc might reciprocally regulate STAT4 expression, accounting for the up-regulation of STAT4 and its target genes in p66Shc overexpressing MEC-1 cells (Figure 1A-1C). To further support this hypothesis we performed a complementary experiment transfecting EBV-B cells with a siRNA targeting the *p66shc* isoform and measured STAT4 by qRT-PCR and immunoblot. A reduction in the mRNA level of *STAT4* was observed in B cells knocked down for p66Shc, which was paralleled by a significant reduction at the protein level (Figure 6A, 6B).

The pro-apoptotic function of p66Shc is correlated with its ability to promote ROS accumulation through two different molecular determinants, one mapping to S36 in the CH2 domain [7] and the second to residues EE132/133 in the cytochrome-*c* binding domain [31]. To assess whether the increase in STAT4 expression in p66Shc-overexpressing cells depends on its pro-oxidant activity we measured the levels of STAT4 in EBV-B cells transfected with vectors encoding either wild-type p66Shc (p66WT) or p66Shc mutants known to impact in its ROS-generating activity, i.e. a S→A substitution at position 36 (p66SA) [7] and E→Q substitutions at positions 132/133 (p66QQ) [31]. p66SA or p66QQ expression resulted in an enhancement in the level of STAT4 comparable to the one observed in the presence of p66WT (Supplementary Figure S4), indicating that the positive regulatory mechanism linking p66Shc to STAT4 expression is independent of its pro-oxidant function.

In summary, we provide evidence of a mechanism responsible for p66Shc expression in B cells that is independent of modifications of the gene promoter and involves the transcription factor STAT4, which is in turn regulated, at least in part, through a feedback loop involving p66Shc. Factors responsible for limiting this loop and molecular determinants of p66Shc involved in the regulation of STAT4 expression still remain to be determined.

By providing cytokines and chemokines, as well as low-affinity BCR ligands, that ensure self-renewal and extended lifespan to the leukemic cells [32], the stromal microenvironment of secondary lymphoid organs and bone marrow plays a major role in the extended survival of CLL B cells. Central to the success of these prosurvival strategies is an effective suppression of the T-cell response. CLL DCs are characterized by an abnormal pattern of cytokine production, which antagonizes the generation of cytotoxic effectors by favouring Th2 polarization and moreover suppress effector function through the production of IL-10 [33, 34]. CLL B cells also contribute to immunosuppression by anergizing T cells and evade killing by establishing dysfuctional immune synapses with CTLs [35]. Some of the immunomodulating cytokines produced by DC or effector T cells in CLL have also the ability to affect leukemic cells, as exemplified by IL-4, which promotes B cell survival [36]. Hence antibody-based immunotherapies involving the combination of IL-12 as adjuvant, which have been proposed as a strategy to correct the Th1/Th2 inbalance in CLL [37], require a thorough assessment of the potential pro-survival effects of this cytokine on the neoplastic cells. Our results provide the first evidence that STAT4, which is central for IL-12R signaling, is expressed at abnormally low levels in CLL B cells. With this as a background we speculate that administration of IL-12 is highly likely to potentiate the antitumor T-cell response without unwanted prosurvival effects on the neoplastic cells, thus highlighting IL-12/STAT4 pathway as a potential target for new therapeutic approaches in CLL.

## MATERIALS AND METHODS

### Cells, plasmids, transfections

MEC-1, EBV-B and human primary B cells were cultured in RPMI-1640 (Sigma-Aldrich, The Woodlands, TX) supplemented with 7.5% bovine calf serum at 37 °C in a humidified atmosphere with 5% CO_2_.

Plasmids included murine Stat4 pRc/CMV (Addgene, Cambridge, MA), pcDNA3.1 (Invitrogen, Carlsbad, CA) and the pcDNA3-p66Shc constructs encoding human wild-type p66Shc or the respective p66ShcSA (S→A substitution at position 36) and p66ShcQQ (EE3→QQ substitutions at positions 132-133) mutants [4].

The esiRNAs used to silence *STAT4* (EHU069141) in human cells, as well as unrelated control RLUC esiRNA (EHURLUC) were purchased from Sigma-Aldrich. *p66shc* silencing in human cells was obtained using the p66Shc-1 siRNA (GenScript Corporation, Piscataway, N.J).

Transfections were carried out using a modification of the DEAE-dextran procedure [38] or by electroporation. Assays were carried out after 24, 48 or 72 h.

Reconstitution of p66Shc expression in CLL B cells was obtained using the Human B-cell Nucleofector Kit (Amaxa Biosystems, Cologne, Germany) according to manufacturer's instructions as previously described [12].

### Patients and healthy donors

Blood samples were collected from 30 patients who satisfied standard morphologic and immunophenotypic criteria for CLL. All cases were obtained by the Hematology and Clinical Immunology Branch, Padua University School of Medicine (Padua, Italy). At the time of the collection, patients had never received treatment. Normal B cells from 9 buffy coats were used as controls for the healthy adult population. Informed consent was obtained from all patients.

B cells (CD19^+^) were purified by negative selection using the RosetteSep Human B-cell enrichment Cocktail (StemCell Technologies, Vancouver, BC), followed by density gradient centrifugation on Lympholite (Cedarlane Laboratories, Burlington, NC).

### Cell treatments and Immunoblotting

EBV-B cells were incubated with 10, 20 or 40 μM LSF (Sigma-Aldrich) for 2, 4 or 16 h after which cells were collected and processed for quantitative RT-PCR or immunoblot analysis. Stimulation with IL-12 was performed following 2 h treatment of EBV-B cells with 20 μM LSF using 2 ng/ml IL-12 (Immunotools, Friesoythe, Germany) for 1 or 2 h.

For immunoblot analyses, cells were lysed in 1% Triton X-100 in 20mM Tris-HCl (pH 8) and 150 mM NaCl (in the presence of a protease inhibitor cocktail, Calbiochem, San Diego, CA). Immunoblot analysis was carried out by chemiluminescence (SuperSignal West Pico Chemiluminescent Substrate kit, Pierce, Waltham, MA) using as primary Abs anti-ShcA (Upstate Biotechnology, Lake Placid, NY), anti-STAT4 (Cell Signaling, Danvers, MA), anti p-STAT4 (Santa Cruz, Santa Cruz, CA), anti-actin (Millipore, Billerica, MA) or anti-Lamin A/C (Cell Signaling) Abs and secondary peroxidase-labeled Abs (Amersham Pharmacia Biotech, Uppsala, Sweden).

### RNA purification, gene expression profiling, and quantitative RT-PCR

Total RNA was extracted from MEC-Ctr and MEC-p66 cells, generated as previously described [12], using the RNeasy Micro Kit (Qiagen, Valencia, CA) and subjected to gene array profile analysis using Affymetrix HuGene-2_0-st-v1 arrays (carried out by the Microarray Unit at Cogentech, Milan, Italy).

Total RNA was extracted from B cells from healthy donors, CLL patients, MEC-1 or EBV-B cells using the RNeasy Plus Mini Kit (Qiagen) and retrotranscribed using the iScript™ cDNA Synthesis Kit (Bio-Rad, Hercules, CA). Three independent reverse transcription reactions were performed on each sample. qRT-PCR was performed in triplicate on each cDNA on 96-well optical PCR plates (Sarstedt, Nümbrecht, Germany) using SSo Fast EvaGreenR SuperMix (Bio-Rad) and a CFX96 Real-Time system (Bio-Rad). Results were processed and analyzed using CFX Manager Version 1.5 software (Bio-Rad). Transcript levels were normalized to housekeeping controls. *HPRT* was used as housekeeping control for all qRT-PCR analyses with the exception of the experiments involving treatment with LSF, for which *ACTB (β-actin)* was used as this treatment resulted in alternations in *HPRT* mRNA levels. The primers used to amplify the cDNA fragments are listed in Supplementary Table S2.

### Reporter gene assay

The human *p66shc* promoter fragments were generated by amplifying the *p66shc* promoter region from DNA extracted from EBV-B cells and ligated into the KpnI/EcoRV sites of the pGL4.17[luc2/Neo] basic vector (Promega, Madison, WI) using the primers listed in Supplementary Table S5. Mutagenesis of the STAT4 binding sites was obtained by Polymerase Incomplete Primer Extension (PIPE) cloning method [40], using the primers listed in Supplemental Table S6.

For normalization of transfection efficiency, the pRL-TK plasmid that expresses Renilla luciferase (Promega) was used. The assays were carried out by transient transfection in EBV-B cells and the luciferase readings were recorded with a Promega Dual-Luciferase Assay System using a Junior LB 9509 Portable Tube Luminometer (Berthold, Harpenden, UK).

### Chromatin immunoprecipitation and *in vitro* binding assay

ChIP assays for analysis of STAT4 occupancy on the *p66shc* promoter was carried out essentially as described by El-Osta and Wolfer [39]. Briefly, 20×10^6^ EBV-B cells were crosslinked with 0.8% formaldehyde for 10 min at room temperature. Cell nuclei were lysed and the lysate was sonicated 10 times for 10 sec to obtain average DNA fragment sizes of 500-1000 bases. After preclearing with 50 μl of protein-A-Sepharose (PAS) slurry (Amersham Pharmacia Biotech, Uppsala, Sweden), immunoprecipitations were carried out overnight using 5 μg of anti-STAT4 or anti-p-STAT4 antibodies, or control IgG, followed by addition of 40 μl of PAS slurry for 2 h to adsorb the protein-DNA complexes. Beads were washed eight-times, eluted in 400μl of buffer (50 mM Tris pH 8.0, 1 mM EDTA, 1% SDS, 50 mM NaHCO_3_), and crosslinking of protein-DNA complexes was reversed by heating at 65°C for 16 h. DNA was extracted with phenol/chloroform and precipitated. The immunoprecipitated DNA fragments were quantitated by qRT-PCR as described above. The primer sets used for the analysis are listed in Supplementary Table S3.

For *in vitro* binding assays the *p66shc* promoter fragments were generated by PCR amplification of genomic DNA extracted from EBV-B cells using the PureLink Genomic DNA mini kit (Invitrogen). The primers used for amplification are listed in Supplementary Table S4. The mutated fragments were generated by PCR reactions using as templates the reporter plasmids with the mutated STAT4 binding sequences (see *Reporter Gene Assay* section). The PCR reactions were performed using the following amplification conditions: 95°C for 3 minutes followed by 40 cycles at 95°C for 30 sec, 60°C for 30 sec, 72°C for 45 sec, and a final elongation at 72°C for 4 min. PCR products were purified with the High Pure PCR Product Purification Kit (Roche Applied Science, Indianapolis, IN) and incubated for 2 h at room temperature with Pierce™ Streptavidin Magnetic Beads. Beads were then incubated overnight with EBV-B nuclear extracts, washed and subjected to SDS-PAGE and immunoblot using anti-STAT4 and anti-Lamin A/C antibodies.

### Flow cytometry and apoptosis assay

Intracellular levels of IL-10, IFN-γ and IL-1β were quantitated on MEC-Ctr and MEC-p66 cells treated with a combination of 100 μg/ml phorbol 12-myristate 13-acetate and 500 ng/ml A23187 for 4 h, then added with 10 μM Brefeldin A and further incubated for 16 h. Cells were then fixed and permeabilized using the Cytofix/Cytoperm plus kit (BD Bioscience, San Jose, CA), stained with anti-human IL-10 (BioLegend, San Diego, CA), IFN-γ (eBioscience, San Diego, CA) or IL-1β (BioLegend) Abs and analyzed using a Guava Easy Cyte (Millipore, Billerica, MA) flow cytometer.

For the apoptosis assays EBV-B cells were incubated with 20 μM LSF for 2 h before stimulation with 2 ng/ml of IL-12 (Immunotools). After 1 h cells were treated with either DMSO or 500 ng/ml A23187 for 15 min at 37°C to induce apoptosis. Apoptosis was measured by flow cytometric analysis of FITC-labeled Annexin-V^+^/PI^−^ (e-Bioscience) stained cells.

### Statistical analysis

Mean values, standard deviations and Student's *t-*test (paired) were calculated using Microsoft Excel (Redmont, WA, USA). The one-way ANOVA with a Tukey's post hoc test was used for experiments in which multiple groups were compared and calculated using the GraphPad Software (La Jolla, CA, USA). A *P*≤0.05 was considered statistically significant.
